# Glycaemic control in Australia and New Zealand before and after the NICE-SUGAR trial: a translational study

**DOI:** 10.1186/cc13030

**Published:** 2013-10-02

**Authors:** Kirsi-Maija Kaukonen, Michael Bailey, David Pilcher, Neil Orford, Simon Finfer, Rinaldo Bellomo

**Affiliations:** 1 Australian and New Zealand Intensive Care Research Centre, Department of Epidemiology and Preventive Medicine, School of Public Health, Monash University, Melbourne, VIC, Australia; 2 Australian & New Zealand Intensive Care Society Centre for Outcome and Resource Evaluation (ANZICS CORE), Ievers Terrace, Melbourne, VIC, Australia; 3 Department of Intensive Care, The Alfred Hospital, Commercial Road, Melbourne, VIC, Australia; 4 Intensive Care Unit, Geelong Hospital, Barwon Health, Geelong, VIC, Australia; 5 The George Institute for Global health, University of Sydney, Sydney, NSW, Australia

## Abstract

**Introduction:**

There is no information on the uptake of Intensive Insulin Therapy (IIT) before the Normoglycemia in Intensive Care Evaluation and Surviving Using Glucose Algorithm Regulation (NICE-SUGAR) trial in Australia and New Zealand (ANZ) and on the bi-national response to the trial, yet such data would provide important information on the evolution of ANZ practice in this field. We aimed to study ANZ glycaemic control before and after the publication of the results of the NICE-SUGAR trial.

**Methods:**

We analysed glucose control in critically ill patients across Australia and New Zealand during a two-year period before and after the publication of the NICE-SUGAR study. We used the mean first day glucose (Glu_1_) (a validated surrogate of ICU glucose control) to define practice. The implementation of an IIT protocol was presumed if the median of Glu_1_ measurements was <6.44 mmol/L for a given ICU. Hypoglycaemia was categorised as severe (glucose ≤2.2 mmol/L) or moderate (glucose ≤3.9 mmol/L).

**Results:**

We studied 49 ICUs and 176,505 patients. No ICU practiced IIT before or after NICE-SUGAR. Overall, Glu_1_ increased from 7.96 (2.95) mmol/L to 8.03 (2.92) mmol/L (*P* <0.0001) after NICE-SUGAR. Similar increases were noted in all patient subgroups studied (surgical, medical, insulin dependent diabetes mellitus, ICU stay >48/<48 hours). The rate of severe and moderate hypoglycaemia before and after NICE-SUGAR study were 0.59% vs. 0.55% (*P* =0.33) and 6.62% vs. 5.68% (*P* <0.0001), respectively. Both crude and adjusted mortalities declined over the study period.

**Conclusions:**

IIT had not been adopted in ANZ before the NICE-SUGAR study and glycaemic control corresponded to that delivered in the control arm of NICE-SUGAR trial. There were only minor changes in practice after the trial toward looser glycaemic control. The rate of moderate hypoglycaemia and mortality decreased along with such changes.

## Introduction

Stress-related hyperglycaemia was traditionally considered a potentially protective physiological reaction to stress [1]. Increased levels of blood glucose, however, are associated with increased morbidity and/or mortality [2-5]. This association triggered randomised controlled trials of intensive insulin therapy from 2001 to 2009 [6-8]. The first single centre trial of intensive insulin therapy (IIT) found a beneficial effect in surgical critically ill patients [6]. The second single centre study [7] in medical patients found benefit only in patients who stayed in ICU for more than three days. The third (Normoglycemia in Intensive Care Evaluation and Surviving Using Glucose Algorithm Regulation (NICE-SUGAR)) multicentre study randomized 6,104 patients from Canada, Australia and New Zealand (ANZ) to intensive or conventional glucose control [8] and found a significant increase in mortality in patients with IIT. A recent meta-analysis confirmed lack of benefit and higher risk of hypoglycaemia with IIT [9]. Hypoglycaemia is strongly associated with increased risk of mortality as is shown for NICE-SUGAR study patients [10].

Recommendations on glucose treatment in critically ill patients have changed in response to new evidence; from IIT (4.4 to 6.1 mmol/L) in surgical critically ill patients [11] to glucose control <8.3 mmol/L in patients with severe sepsis [12,13] to looser control of glucose levels while awaiting for more evidence [14] and finally to moderate glucose control (<10 mmol/L) for all critically ill patients [15,16].

Two important issues, among others, however, remain to be addressed. The first is the representativeness of existing evidence on glycaemic control in ANZ ICUs before NICE-SUGAR. An inception cohort study with 29 ICUs and 939 patients described glucose control practice and glycaemic control in ANZ before the NICE-SUGAR study [17]. However, it is unknown to what degree these patients were representative of ANZ practice. The second is whether changes in available data have been translated into practice and have affected glycaemic control in ANZ. The first is important in supporting the robustness of the findings of the NICE-SUGAR study; the second in defining how evidence might be translated into practice at a bi-national level after a pivotal trial.

The Australian and New Zealand Intensive Care Society Adult Patient Database (ANZICS APD) is a high quality database, which routinely collects lowest and highest glucose values during the first 24 h in ICU [18]. In a study involving >8,000 patients and close to 200,000 glucose measurements, the average glucose levels on Day 1 was found to be an excellent surrogate of overall ICU glucose control with a mean difference of only 0.17 mmol/L [19].

Accordingly, we used data from the ANZICS APD to test the hypotheses that 1.) glycaemic control practice in ANZ at the time of the NICE-SUGAR trial was equivalent to that prescribed to the trial control group and 2.) the NICE-SUGAR trial results have been translated into practice with the mean glucose level of 8.0 mmol/L.

## Materials and methods

We conducted a retrospective analysis of the prospectively collected Australian and New Zealand Intensive Care Society Adult Patient Database [18]. The ANZICS-APD is an established voluntary bi-national database run by the ANZICS Centre for Outcome and Resource Evaluation (CORE) which contains information on 80% of the ICU admissions in Australia and New Zealand [20]. Non-contributing sites are predominantly small rural and private hospitals. The information collection includes clinical and physiological information as well as outcome data for routine quality surveillance [21]. Data are collected under the Quality Assurance Legislation of the Commonwealth of Australia (Part VC Health Insurance Act 1973, Commonwealth of Australia). Access to the data was granted by the ANZICS-CORE Management Committee in accordance with standing protocols. The study was approved by The Alfred Health Human Ethics Committee.

### Glucose control

The highest and the lowest blood glucose measurement during the first 24 hours after ICU admission are recorded into APD. These two values were used to define all glycaemic indices in the study. The mean value of these measurements (Glu_1_) has been shown to act as an accurate surrogate of glucose control for the whole duration of ICU stay [19]. Accordingly, we used the Glu_1_ value as a surrogate of glucose control for each patient during their ICU stay. The first day lowest glucose measurement was used to define the rate of hypoglycaemia. The rate of hypoglycaemia was calculated by dividing the number of patients with hypoglycaemia by all patients eligible for assessment. Severe hypoglycaemia was defined as glucose value ≤2.2 mmol/L and moderate hypoglycaemia as a glucose value ≤3.9 mmol/L [6-8].

The glucose control for each ICU was defined by calculating the median value of all patients’ Glu_1_ values. The implementation of tight glucose control in clinical practice for the ICU was presumed if the median of the Glu_1_ measurement was <6.44 mmol/L. The value of 6.44 mmol/L was chosen as it represents the upper limit of 95% confidence interval of the observed time-averaged mean glucose value in the IIT arm of NICE-SUGAR [8].

For outcome analysis, the ICUs were divided into quartiles according to the extent of change in their Glu_1_ values. The first quartile (Q1) represents ICUs with the least increase in the Glu_1_ value and the fourth quartile (Q4) represents ICUs with the highest increase.

### Selection of ICUs and time period

All ICUs providing data to APD were potentially eligible for the study. We ensured the representativeness of the patient population for a given ICU by excluding ICUs that had not annually provided at least 80% of the first day glucose measurements for their patients for the duration of the entire study period. As the number of ICUs fulfilling this criterion during early years of millennium was low, we could not reliably study the translation of the first two large randomised controlled trials (RCTs) into practice. Therefore, we restricted the study period around the latest large RCT (NICE-SUGAR [8]). NICE-SUGAR was published 6 March 2009 and, accordingly, we collected patients for two years before NICE-SUGAR (from 1February 2007 to 31 January 2009) and two years after (from 1 June 2009 to 31 May 2011). We also studied time-related changes by splitting the before and after NICE-SUGAR study periods into six-month periods.

### Patients

We studied all patients treated in the participating ICUs during the study period. The results are presented also in patient subgroups as reported in previous large RCTs by admission diagnosis: medical [7] or surgical [6] and insulin-dependent diabetes mellitus (IDDM) reported at admission [22]. We also analysed patients with an ICU stay >48 hours [7,8].

### Statistical analysis

Data are presented as mean (standard deviation, SD) or median (interquartile range, IQR). The variables are compared using Student’s *t*-test, Wilcoxon Rank Sum test or Chi-square test depending on the nature and distribution of the variable to be analysed. Hospital mortality was adjusted by Acute Physiology and Chronic Health Evaluation III (APACHE III) risk of death. We performed logistic regression analyses for both crude and adjusted hospital mortality with the first six-month period as the reference. To further account for temporal changes over time, segmented regression analysis of interrupted time series was performed on the number of events occurring each month before and after NICE-SUGAR, with the mean APACHE III score for all patients within each monthly period also included as a covariate to indirectly account for patient severity. Segmented regression analysis was performed by fitting a line before NICE-SUGAR, a separate line after NICE-SUGAR, and a binomial term for before/after to determine if there is significant vertical shift between the two periods. All data were analysed by SAS Version 9.2 (SAS Institute Inc., Cary, NC, USA). Two-tailed *P*-value of 0.01 was considered as statistically significant.

## Results

Of 132 ICUs providing data to the APD, 49 fulfilled the selection criteria and of them, 9 (18%) participated in NICE-SUGAR. During the study period, 176,505 patients were admitted to these ICUs. Patient characteristics before and after NICE-SUGAR study are presented in the Additional file 1: Table A1.

### Glucose control in ICUs

No ICU applied IIT in their clinical practice either before or after the publication of NICE-SUGAR study. Overall, 28 of the 49 ICUs had an increase in the median Glu_1_ value. Of the nine ICUs that had participated in NICE-SUGAR, five slightly loosened their glucose control and four ICUs slightly tightened it after the publication of results. The ICUs in NICE-SUGAR study had slightly looser glucose control before the study than the ICUs not participating in NICE-SUGAR. Overall, both ICU types (participating and not participating in NICE-SUGAR) loosened their glucose control after the publication of the study results, the increase being slightly higher in ICUs participating to NICE-SUGAR (Table 1).

**Table 1:** Median of the mean first day glucose measurement according to different types of Intensive Care Units

**Hospital level**	**Before NICE-SUGAR**	**After NICE-SUGAR**	***P***
Rural ICUs	7.15 (6.0 to 8.6)	7.15 (6.0 to 8.9)	0.52
Metropolitan ICUs	7.65 (6.4 to 9.35)	7.55 (6.3 to 9.35)	0.07
Tertiary ICUs	7.55 (6.4 to 8.8)	7.70 (6.55 to 9.05)	<0.0001
Private ICUs	7.45 (6.4 to 8.5)	7.40 (6.35 to 8.45)	0.004
NICE-SUGAR ICUs	7.55 (6.35 to 8.9)	7.65 (6.45 to 9.05)	<0.0001
No NICE-SUGAR ICUs	7.50 (6.4 to 8.8)	7.55 (6.45 to 8.9)	<0.0001

ICU, intensive care unit; IQR, median; NICE-SUGAR, Normoglycemia in Intensive Care Evaluation and Surviving Using Glucose Algorithm Regulation.

Rural ICUs had the tightest glucose control and metropolitan ICUs the loosest glucose control before NICE-SUGAR study (Table 1). After the NICE-SUGAR study, tertiary ICUs loosened their glucose control, rural or metropolitan ICUs made no changes (Table 1). Surprisingly, private ICUs tightened their glucose control after NICE-SUGAR study. However, all these changes were minute in extent and unlikely to be clinically significant.

### Glucose control at patient level

The mean (SD) value of Glu_1_ increased from 7.96 (2.95) mmol/L to 8.03 (2.92) mmol/L (*P* <0.0001) after NICE-SUGAR in all patients (Table 2). Statistically significant increases were noted in all patient subpopulations studied: surgical, medical, IDDM, no-IDDM, ICU stay more or less than 48 hours (Table 2).

**Table 2:** **Mean first day glucose (Glu**_**1**_**, in mmol/L) in patients during the two study periods**

	**Before NICE-SUGAR**	**After NICE-SUGAR**	***P***
All patients	7.96 (2.95)	8.03 (2.92)	<0.0001
Surgical patients	7.8 (2.2)	7.9 (2.2)	<0.0001
Medical patients	8.1 (3.6)	8.2 (3.5)	0.002
IDDM patients	10.8 (5.3)	11.2 (5.4)	<0.0001
Non-IDDM patients	7.8 (2.7)	7.8 (2.7)	<0.0001
Patients with >48 h ICU stay	8.2 (3.0)	8.3 (2.9)	<0.0001
Patients with <48 h ICU stay	7.8 (2.9)	7.9 (2.9)	<0.0001

All numbers are mean (SD). IDDM, insulin-dependent diabetes mellitus; NICE-SUGAR, Normoglycemia in Intensive Care Evaluation and Surviving Using Glucose Algorithm Regulation.

### Glucose control over time

**Figure 1:**
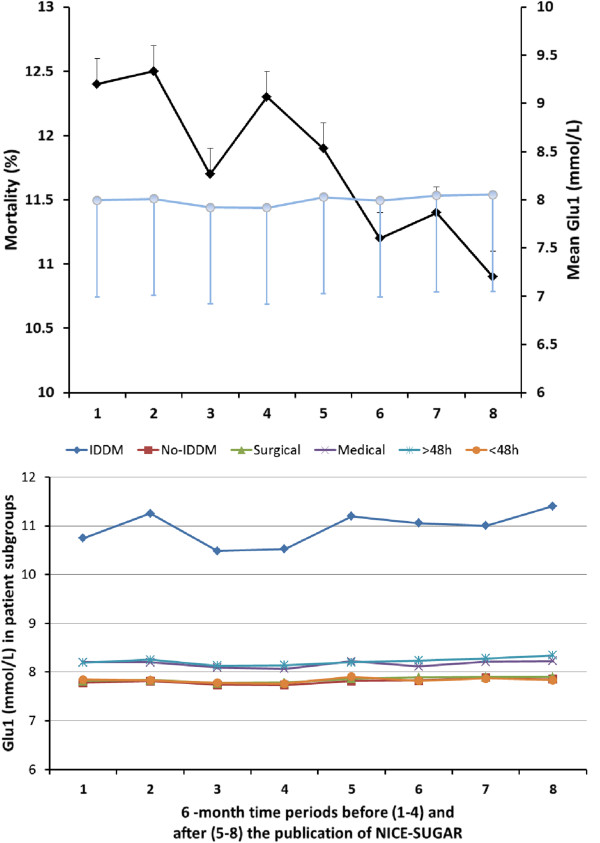
**Glu**_**1**_**, mortality and Glu**_**1 **_**in subgroups over time before and after NICE-SUGAR.** Mean first day glucose (SE, Glu_1_, blue circles) in all patients with crude hospital mortality (SE, black squares) in four consecutive six-month periods before (periods 1 to 4) and after (periods 5 to 8) Normoglycemia in Intensive Care Evaluation and Surviving Using Glucose Algorithm Regulation (NICE-SUGAR) study (upper panel). Mean Glu_1_ values in patient subgroups (surgical/medical, IDDM/no IDDM, ICU stay >48 h/ICU stay <48 h) in the same six-month periods (lower panel). IDDM, insulin-dependent diabetes mellitus.

The means of Glu_1_ values over consecutive six-month periods before and after the NICE-SUGAR study in all patients as well as in patient subgroups (surgical, medical, IDDM/no-IDDM, ICU stay >48 h/<48 h) are presented in Figure 1.

### Severe and moderate hypoglycaemia

The rates of severe hypoglycaemia were 0.59% and 0.55% (*P* = 0.33) in all patients before and after NICE-SUGAR publication, respectively (Table 3). There were no statistically significant differences in the rates of severe hypoglycaemia before and after NICE-SUGAR publication in any patient subgroup (Table 3). The rate of moderate hypoglycaemia decreased by 14.2% in the overall patient population as well as in patient subgroups, with the exception of medical patients or patients with IDDM (Table 3). Moderate hypoglycaemia rate decreased in ICUs not participating into NICE-SUGAR but not in ICUs conducting the study (Table 3). In rural and metropolitan ICUs, the rate of moderate hypoglycaemia remained the same, whereas in tertiary and private ICUs the rates decreased from before to after NICE-SUGAR (Table 3).

**Table 3:** Rate of moderate and severe hypoglycaemia during the first 24 hours after ICU admission

	**Severe hypoglycaemia**			**Moderate hypoglycaemia**		
	**Glucose ≤2.2 mmol/L**			**Glucose ≤3.9 mmol/L**		
	**Before NICE-SUGAR**	**After NICE-SUGAR**	***P***	**Before NICE-SUGAR**	**After NICE-SUGAR**	***P***
All patients	0.59% (449/76,600)	0.55% (489/88,896)	0.33	6.62% (5,076/76,600)	5.68% (5,051/88,896)	<0.0001
Surgical patients	0.26% (106/40,847)	0.23% (109/47,670)	0.35	4.48% (1,828/40,847)	3.17% (1,511/47,670)	<0.0001
Medical patients	0.96% (339/35,433)	0.93% (380/41,077)	0.65	9.12% (3,230/35,433)	8.61% (3,531/41,077)	0.012
IDDM patients	1.72% (43/2,499)	1.55% (38/2,454)	0.63	14.21% (355/2,499)	13.20% (324/2,454)	0.3
No-IDDM patients	0.57% (286/49,995)	0.52% (310/59,966)	0.22	6.79% (3,394/49,995)	5.64% (3,385/59,966)	<0.0001
Patients with >48 h ICU stay	0.63% (201/32,089)	0.64% (241/37,702)	0.83	7.85% (2,520/32,089)	6.87% (2,591/37,702)	<0.0001
Patients with <48 h ICU stay	0.56% (248/44,488)	0.48% (248/51,169)	0.12	5.74% (2,554/44,488)	4.80% (2,458/51,169)	<0.0001
NICE-SUGAR ICU	0.60% (100/16,794)	0.57% (111/19,346)	0.79	5.69% (955/16,794)	5.62% (1,087/19,346)	0.78
Not NICE-SUGAR ICU	0.58% (349/59,806)	0.54% (378/69,550)	0.34	6.89% (4,121/59,806)	5.70% (3,964/69,550)	<0.0001
Hospital level						
Rural	0.66% (31/4,708)	0.6% (27/4,506)	0.72	6.65% (313/4,708)	6.88% (310/4,506)	0.66
Metropolitan	0.73% (83/11,414)	0.79% (104/13,128)	0.56	6.59% (752/11,414)	6.51% (854/13,128)	0.79
Tertiary	0.66% (301/45,756)	0.61% (319/52,634)	0.31	7.65% (3,500/45,756)	6.5% (3,421/52,364)	<0.0001
Private	0.23% (34/14,722)	0.21% (39/18,628)	0.68	3.47% (511/14,722)	2.5% (466/18,628)	<0.0001

The data are given as percentages (number of patients with the event/number of patients with data available). ICU, Intensive Care Unit; IDDM, Insulin dependent diabetes mellitus; NICE-SUGAR, Normoglycemia in Intensive Care Evaluation and Surviving Using Glucose Algorithm Regulation.

### Diabetic patients

The patients with IDDM had the highest Glu_1_ values both before and after NICE-SUGAR study as well as and the highest increase in Glu_1_ (Figure 1, Table 2). The rate of severe and moderate hypoglycaemia in patients with IDDM was more than two-fold that in other patient subgroups (Table 3). Patients with IDDM were the only patient subgroup where crude mortality did not decrease from before to after NICE-SUGAR (Table 4).

**Table 4:** Unadjusted hospital mortality in patient subgroups

	**Before NICE-SUGAR**	**After NICE-SUGAR**	***P***
	**n = 82,740**	**n = 93,765**	
Medical patients*	19.8% (7,569)	18.5% (8,020)	<0.0001
Surgical patients*	5.4% (2,345)	4.91% (2,427)	0.0008
IDDM patients*	13.9% (360)	11.86% (297)	0.029
No-IDDM patients*	12.07% (6,566)	10.93% (6,943)	<0.0001
Patients with >48 h ICU stay*	16.58% (5,489)	15.32% (5,897)	<0.0001
Patients with <48 h ICU stay*	9.06% (4,496)	8.32% (4,596)	<0.0001

*percentage (number of events). NICE-SUGAR, Normoglycemia in Intensive Care Evaluation and Surviving Using Glucose Algorithm Regulation.

### Outcomes of patients

**Figure 2:**
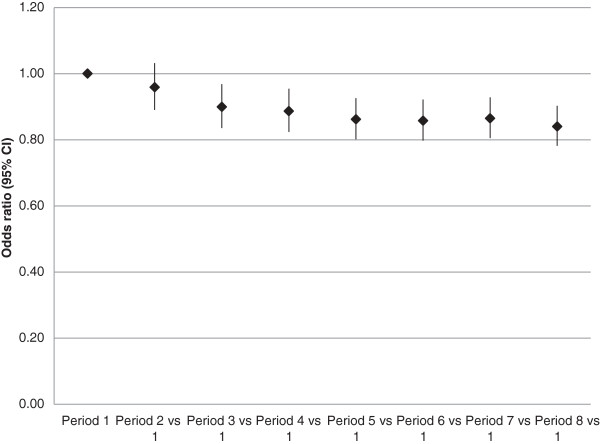
**Adjusted mortality over time before and after NICE-SUGAR.** Odds ratios (95% confidence intervals) for hospital mortality in all patients during four consecutive six-month periods before (periods 1 to 4) and after (periods 5 to 8) NICE-SUGAR study (*P* <0.0001). NICE-SUGAR, Normoglycemia in Intensive Care Evaluation and Surviving Using Glucose Algorithm Regulation.

ICU length of stay increased after NICE-SUGAR, but hospital length of stay decreased in all patients (Table 5). The outcomes of all patients before and after NICE-SUGAR study are presented in Table 5. Unadjusted mortality declined over time during the whole study period (Table 5, Figure 1). There was a decrease in unadjusted mortality in all patient subgroups before vs. after NICE-SUGAR periods. It was statistically significant in all patient subgroups except for IDDM patients (Table 4). The adjusted mortality decreased during the whole study period (Figure 2).

**Table 5:** Outcome of patients

	**Before NICE-SUGAR**	**After NICE-SUGAR**	***P***
	**n = 82,740**	**n = 93,765**	
ICU LOS (hours, IQR)	39.7 (21.1 to 80.1)	40.8 (21.5 to 84.3)	<0.0001
Hospital LOS (days, IQR)	9.7 (5.4 to 18.2)	9.3 (5.3 to 17.8)	0.002
Hospital outcome: home*	68% (56,673)	69% (64,547)	0.12
ICU mortality*	8% (6,480)	7% (6,728)	<0.0001
Hospital mortality*	12% (9,989)	11% (10,494)	<0.0001

*percentage (number of events); ICU, intensive care unit; LOS, length of stay; NICE-SUGAR, Normoglycemia in Intensive Care Evaluation and Surviving Using Glucose Algorithm Regulation.

**Figure 3:**
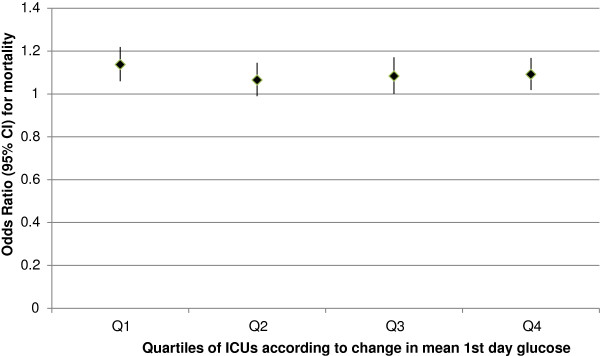
**Adjusted mortality in quartiles of ICUs according to change in Glu**_**1**_**.** Odds ratios (95% confidence intervals) for mortality in quartiles of ICUs according to changes in mean first day glucose values (Q1 denotes ICUs with the smallest change, Q4 denotes ICUs with the highest increase). *P* <0.001 for Q1, *P* ns (>0.01) for Q2 to 4.

In quartiles according to the change in mean Glu_1_, the unadjusted mortality decreased to some degree in all quartiles. However, after adjustment for patient severity, only patients from ICUs with the smallest increase in Glu_1_ (Q1) displayed a statistically significant decline in mortality (Figure 3).

**Figure 4:**
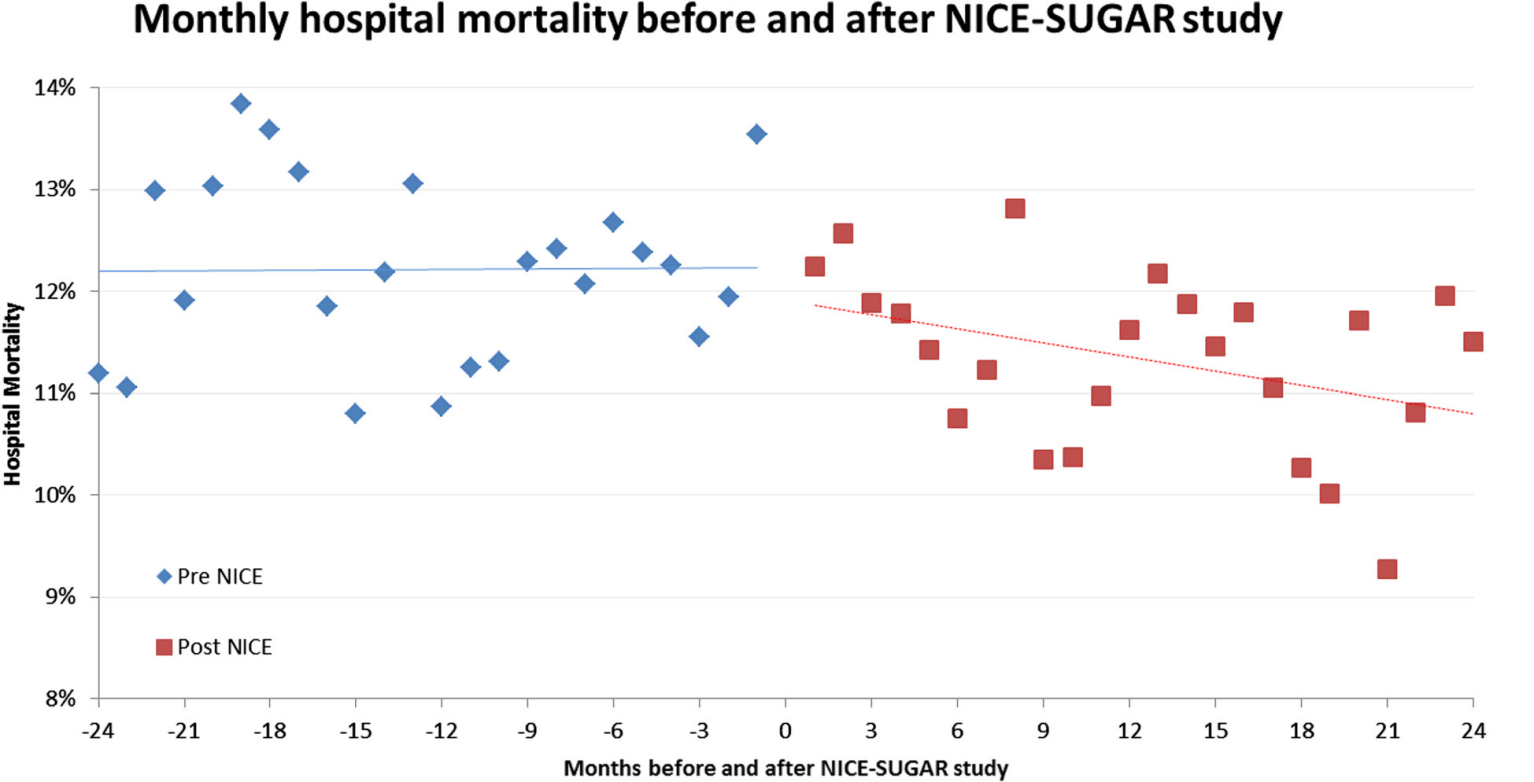
**Interrupted time series analysis of mortality before and after NICE-SUGAR.** Interrupted time series analysis of mortality before and after NICE-SUGAR study. There was no significant vertical shift between pre and post periods (*P* = 0.29). NICE-SUGAR, Normoglycemia in Intensive Care Evaluation and Surviving Using Glucose Algorithm Regulation.

In the segmented time series analysis of hospital mortality, there was no change between before and after NICE-SUGAR periods (Figure 4, *P* = 0.29).

## Discussion

### Key findings

We studied clinical practice before and after a key multicentre randomised controlled study of glucose control in >170,000 patients from 49 ICUs in ANZ. Our findings confirm previously published survey data that IIT was not practiced in ANZ prior to NICE-SUGAR. Glycaemic control in all patients before NICE-SUGAR corresponded to the control group of the trial. We found that only minute changes in glycaemic control toward looser control followed the publication of the study. The change in glycaemic control applied similarly to all patient subgroups except for IDDM patients. The rate of severe hypoglycaemia remained unchanged, whereas the rate of moderate hypoglycaemia decreased by 14% after NICE-SUGAR. IDDM patients had highest Glu_1−_values, highest increase in Glu_1_ values, and highest rates of moderate and severe hypoglycaemia before and after NICE-SUGAR. The adjusted mortality decreased over the whole study period.

### Relationship to previous studies

The mean Glu_1_ for all patients in our study before NICE-SUGAR was 7.96 mmol/L. This is in accordance with the time-averaged mean glucose value of 8.0 mmol/L in the control group of NICE-SUGAR [8]. In each subgroup, glycaemic control approximated to 8 mmol/L, except for patients with IDDM. As the number of IDDM patients represented only 3% of all patients before NICE-SUGAR, their impact on overall glycaemic control was small.

The quality requirement for data supply for individual ICUs was stringent. As the possible bias caused by missing data is of minor concern, it is reasonable to argue that the results of glycaemia in ICUs reflect the true practice of ANZ ICUs at that time. According to our results, IIT was not standard practice in ANZ as none of the ICUs had their median Glu_1_ below the upper limit of the 95% confidence interval of glycaemia reported in the IIT arm of NICE-SUGAR [8]. In an observational study conducted in ANZ ICUs and published in 2003, 41% of ICUs reported having implemented tight glucose control in their clinical practice for at least some patient subgroups [17]. Simultaneous observational data, however, displayed a mean first day glucose value of 8.7 mmol/L [23] suggesting that most ICUs in ANZ did not, in fact, practice IIT.

A nationwide translational study in Germany showed that 65% of ICUs perceived having tight glucose control in practice for patients with sepsis, but only 6% of patients were observed to be in the tight glycaemic range [24]. There are no previous reports of national level translation of glycaemic control recommendations into practice in critically ill patients. Previous studies have shown that the translation of study knowledge of simple, cheap and well-recognized therapies may be poor [25-27].

The loosened glycaemic control after NICE-SUGAR was reflected by the rates of moderate hypoglycaemia. The rates of moderate hypoglycaemia were roughly one-third lower than the 15.8% seen in the control arm of NICE-SUGAR [10]. A partial explanation for this difference may be that we had only first day glucose measurements available. The median time of occurrence of moderate hypoglycaemia in NICE-SUGAR was one day, indicating that half of moderate hypoglycaemia occurred after the first day [10]. There is no validation yet that the incidence of hypoglycaemia on Day 1 is a robust surrogate for the overall incidence of hypoglycaemia.

The rate of severe hypoglycaemia remained unchanged in the overall patient population as well as in the subgroups despite looser glycaemic control after NICE-SUGAR. The rates of severe hypoglycaemia in the control arms of the three RCTs were 0.8% [6], 3.1% [7] and 0.5% [8]. The rate in our unselected patient population approximated that of the first Leuven and NICE-SUGAR studies. In medical patients, our clinical data showed much lower incidence of severe hypoglycaemia than the rate in the second Leuven study which included medical patients only. However, once again, our data are limited to Day 1 after ICU admission.

The patients with IDDM had higher Glu_1_ than patients in any other subgroup, both before and after NICE-SUGAR. In the second Leuven or NICE-SUGAR studies, the mortality in diabetic patients was not different between the treatment groups [7,8]. In a large observational study, hyperglycaemia was associated with increased mortality in non-diabetic patients whereas in diabetic patients the risk was much lower, suggesting that patients with IDDM may have a different biological response to hyperglycaemia [22] or that in patients with DM hyperglycaemia is indicative of a lesser degree of stress of illness severity. Interestingly, the Glu_1_ values in our IDDM patients’ values approximated those in the above observational study suggesting that there might be greater difficulty in controlling glycaemia in diabetic patients or that tight glycaemic control is less vigorously pursued or both [22].

IDDM patients had the highest Glu_1_ of all patient subgroups along with the highest rate of both severe and moderate hypoglycaemia, reflecting the highest variability in glucose values of all patient subgroups. Although hyperglycaemia does not seem to be as harmful in diabetics as in non-diabetic patients [22], higher variability of blood glucose values has been associated with increased risk of mortality in overall critically ill patients [28]. Interestingly, we could not demonstrate a decrease in mortality in IDDM patients. Finally, overall mortality decreased during the study period, even when adjusted for disease severity. As the change towards looser glycaemic control in the overall population was clinically insignificant, it is unlikely to be an explanation for reduced mortality.

### Implications of study findings

The glycaemic control before NICE-SUGAR in ANZ represented clinical practice at that time. It closely followed the glycaemic control achieved in the control arm of NICE-SUGAR enforcing the generalizability of NICE-SUGAR study results to ANZ and justifying the choice of the specific range of glycaemic level apply to the control arm of NICE-SUGAR.

Glycaemic control before NICE-SUGAR was not in the range of IIT in any of the ANZ ICUs included in our study. This is strong evidence that before the NICE-SUGAR study, ICUs in ANZ were not practicing glucose control according to recommendations at the time or failed to achieve targeted glucose control in their patients. The ICUs participating NICE-SUGAR loosened their glucose control practice after the publication of the study results to greater degree than the ICUs not participating in the trial. Overall, the translation of study results into practice seemed to be more prominent in academic ICUs as well as ICUs participating in the trial. This suggests that engaging ICUs in clinical research could facilitate translation of research into practice.

The extent of change in glycaemic control was clinically insignificant. It was, however, reflected by the lower incidence of moderate hypoglycaemia. As hypoglycaemia have been strongly associated with increased mortality, this change appears desirable.

Patients with IDDM were outliers in glycaemic control and hypoglycaemia rate. In addition, IDDM patients were the only subgroup in whom the mortality did not decrease over the study period. The recommendations for glycaemic control in critically ill patients do not separately address glycaemic control in IDDM patients. As this subgroup of patients seems to be highly different from the overall critically ill patient population as well as other patient subgroups, a different approach to their glycaemic control may be needed. If further studies on glycaemic control in critically ill patients are to be conducted, appropriate identification and stratified randomisation for this patient subgroup would be needed.

The observational period of our study was nearly five years leading to a large sample size of more than 170,000 patients. Accordingly, even for small changes in the numerical values between the study periods, statistical significance was easily achieved. In order to increase the robustness of our findings, a reduced *P*-value of 0.01 was chosen to indicate statistical significance. However, even with this conservative statistical approach, the clinical importance of some of the statistically significant changes seen remains unclear.

### Strengths and weaknesses

Our study has several strengths. The data in the APD are prospectively collected for routine quality control purposes and, therefore, they are unlikely to be biased. We ensured the representativeness of the glycaemic control for each ICU by selecting only the ICUs that provided >80% of the first day glucose values for their patients. Some limitations should be noted, however. The completeness of first day glucose values for ICUs was low in the data collected 12 years ago. Thus, we could not analyse the effect of the first Leuven trial on clinical practice. It can be postulated that the greatest changes in clinical practice would have been before and after the publication of the first pivotal RCT with tight glucose control. However, by the time NICE-SUGAR was conducted, such changes (if they had occurred) had clearly dissipated. Secondly, we did not have glucose measurements beyond the first 24 hours of the ICU stay in the database. The first day mean glucose values, however, have been shown to have high accuracy in predicting the glucose control of the whole ICU stay [19]. Third, our assessment of hypoglycaemia using Day 1 data is not validated. However, although the absolute overall incidence of hypoglycaemia may be inaccurate, the change from one period to another using the same assessment methodology is likely to represent a real change in its incidence within ANZ.

## Conclusions

The treatment of the control arm in NICE-SUGAR was an almost exact replica of the standard of care at the time of planning and execution of the study. The changes in overall glucose control before and after NICE-SUGAR were minor and toward looser glucose control. These changes were greater in trial ICUs. The incidence of moderate hypoglycaemia, but not severe hypoglycaemia, on Day 1 decreased with such loosening of glucose control. The results of intensive care treatment continued to improve over time, as measured by hospital mortality. These findings support the notion that, at this stage, the glucose target prescribed in the control arm of NICE represents an acceptable standard of glycaemic control.

## Key messages

•Despite recommendations current at the time, intensive insulin therapy was not adopted in ANZ intensive care units before the publication of NICE-SUGAR study.

•Glucose control before NICE-SUGAR was in the glycaemic band of the control group of NICE-SUGAR confirming that the trial reflected current ANZ practice at the time.

•The changes in clinical practice were small after the result of publication of NICE-SUGAR study and toward looser glycaemic control.

•The change to looser glycaemic control was associated with a decrease in the incidence of moderate hypoglycaemia but not severe hypoglycaemia.

•Irrespective of glycaemic management, hospital mortality and hospital length of stay continued to decrease over the whole study period.

## Abbreviations

ANZ: Australia and New Zealand; ANZICS: Australian and New Zealand Intensive Care Society CORE Centre for Outcomes and Resource Evaluation; Glu1: Mean glucose value of the first 24 hours after ICU admission; ICU: Intensive care unit; IDDM: Insulin-dependent diabetes mellitus; IIT: Intensive insulin therapy; IQR: Interquartile range; Q: Quartile; RCT: Randomised controlled trial; SD: Standard deviation.

## Competing interests

The authors declare that they have no competing interests.

## Authors’ contributions

KMK contributed to study conception and design, analysis and interpretation of data, drafting of the article, and final approval of the version to be published. MB made a substantial contribution to study conception and design, acquisition of data, and statistical analysis of data, critical revision of the manuscript for important intellectual content and final approval of the version to be published. DP contributed to interpretation of data, critical revision of the manuscript for important intellectual content and final approval of the version to be published. NO contributed to the conception and design of the study, made a substantial contribution to the analysis and interpretation of the data, critical revision of the manuscript for important intellectual content and final approval of the version to be published. SF made a substantial contribution to the conception and design of the study, contribution to the interpretation of data, critical revision of the manuscript for important intellectual content and final approval of the version to be published. RB made a substantial contribution to conception and design of the study, analysis plan and interpretation of data, critical revision of the manuscript for important intellectual content and final approval of the version to be published. All authors read and approved the final manuscript.

## Supplementary Material

Additional file 1: Table A1 Patient characteristics and outcomes in all patients before and after NICE-SUGAR study publication. Click here for file
