# Supplementary material for: Glycaemic control in Australia and New Zealand before and after the NICE-SUGAR trial: a translational study
Source: Crit Care. 2013 Oct 2;17(5):R215. doi: 10.1186/cc13030 (PMC4056083; doi:10.1186/cc13030)
Supplement: Additional file 1: Table A1 — Patient characteristics and outcomes in all patients before and after NICE-SUGAR study publication. [file cc13030-S1.docx]

Additional files:

Table A1: Patient characteristics and outcomes in all patients before and after NICE-SUGAR study publication.

|  | **Reported** | **Data available (n)** | **Before NICE-SUGAR** | **Data available (n)** | **After NICE-SUGAR** | **P value** |
| --- | --- | --- | --- | --- | --- | --- |
| No of patients |  | 82740 |  | 93765 |  |  |
| Age | Mean (SD) | 82740 | 59.7 (19) | 93765 | 60.0 (19) | 0.003 |
| Male Gender | Percent (n) | 82740 | 59% (49207) | 93765 | 59% (54922) | 0.0001 |
| APACHE II Score | Mean (SD) | 77500 | 16.24 (7.9) | 92221 | 15.91 (7.52) | <0.0001 |
| APACHE III Score | Median (IQR) | 77698 | 51 (37-70) | 81618 | 51 (37-69) | 0.005 |
| Mechanical ventilation | Percent (n) | 82683 | 48% (39313) | 93764 | 46% (43509) | <0.0001 |
| Elective surgery | Percent (n) | 81591 | 41% (33356) | 93014 | 41% (37744) | 0.2 |
| Surgical admission | Percent (n) | 81635 | 53% (43465) | 92859 | 53% (49462) | 0.92 |
| Cardiac Surgery | Percent (n) | 81635 | 20% (16534) | 92859 | 19% (17601) | <0.0001 |
| IDDM | Percent (n) | 56967 | 4.5% (2589) | 66044 | 3.8% (2505) | <0.0001 |
| Highest Glucose during first 24 hours (mmol/L) | Median (IQR) | 78754 | 8.7 (7.1-10.7) | 90256 | 8.8 (7.2-10.9) | <0.0001 |
| Lowest Glucose during first 24 hours (mmol/L) | Median (IQR) | 76600 | 6 (5.1-7.1) | 88896 | 6.1 (5.2-7.2) | <0.0001 |
| **Hospital Admission Source:**  Chronic Care | Percent (n) | 82740 | 1% (1101) | 93765 | 1% (1133) | 0.022 |
| Home | Percent (n) | 82740 | 73% (60413) | 93765 | 76% (71404) | <0.0001 |
| Other Hospital | Percent (n) | 82740 | 18% (14574) | 93765 | 17% (15980) | 0.002 |
| Other ICU | Percent (n) | 82740 | 1% (862) | 93765 | 1% (1044) | 0.15 |
| **ICU Admission Source:**  Emergency | Percent (n) | 82740 | 26% (21199) | 93765 | 26% (24066) | 0.83 |
| Operating Theatre | Percent (n) | 82740 | 52% (43185) | 93765 | 52% (48916) | 0.92 |
| Other ICU | Percent (n) | 82740 | 9% (7306) | 93765 | 9% (8017) | 0.037 |
| Ward | Percent (n) | 82740 | 13% (10966) | 93765 | 14% (12702) | 0.07 |
| ICU Length of Stay (hours) | Median (IQR) | 82711 | 39.67 (21.13-80.1) | 93734 | 40.75 (21.5-84.33) | <0.0001 |
| **Hospital outcome:**  Home | Percent (n) | 82740 | 68% (56673) | 93765 | 69% (64547) | 0.12 |
| Other Hospital | Percent (n) | 82740 | 9% (7598) | 93765 | 9% (8536) | 0.56 |
| Rehabilitation | Percent (n) | 82740 | 9% (7372) | 93765 | 10% (9082) | <0.0001 |
| Hospital Length of Stay (days) | Median (IQR) | 82093 | 9.68 (5.44-18.21) | 93230 | 9.3 (5.28-17.84) | 0.002 |
| Hospital Mortality | Percent (n) | 81632 | 12% (9989) | 92659 | 11% (10494) | <0.0001 |

APACHE Acute Physiology And Chronic Health Evaluation, IDDM Insulin-Dependent Diabetes Mellitus, ICU Intensive Care Unit
